# IDP-EDL: enhancing intrinsically disordered protein prediction by combining protein language model and ensemble deep learning

**DOI:** 10.1093/bib/bbaf182

**Published:** 2025-04-21

**Authors:** Junxi Xie, Xiaopeng Jin, Hang Wei, SaiSai Sun, Yumeng Liu

**Affiliations:** College of Big Data and Internet, Shenzhen Technology University, 3002 Lantian Road, Pingshan District, Shenzhen, Guangdong 518118, China; College of Big Data and Internet, Shenzhen Technology University, 3002 Lantian Road, Pingshan District, Shenzhen, Guangdong 518118, China; School of Computer Science and Technology, Xidian University, South Campus: 266 Xinglong Section of Xifeng Road, Xi’an, Shaanxi 710126, North Campus: No. 2 South Taibai Road, Xi’an, Shaanxi 710071, China; School of Computer Science and Technology, Xidian University, South Campus: 266 Xinglong Section of Xifeng Road, Xi’an, Shaanxi 710126, North Campus: No. 2 South Taibai Road, Xi’an, Shaanxi 710071, China; College of Big Data and Internet, Shenzhen Technology University, 3002 Lantian Road, Pingshan District, Shenzhen, Guangdong 518118, China

**Keywords:** intrinsically disordered regions, protein language model, task-specific fine-tuning, ensemble deep learning

## Abstract

Identification of intrinsically disordered regions (IDRs) in proteins is essential for understanding fundamental cellular processes. The IDRs can be divided into long disordered regions (LDRs) and short disordered regions (SDRs) according to their lengths. In previous studies, most computational methods ignored the differences between LDRs and SDRs, and therefore failed to capture the different patterns of LDRs and SDRs. In this study, we propose IDP-EDL, an ensemble of three predictors. The component predictors were first built based on pretrained protein language model and applied task-specific fine-tuning for short, long, and generic disordered regions. A meta predictor was then trained to integrate three task-specific predictors into the final predictor. The results of experiments show that task-specific supervised fine-tuning can capture the different features of LDRs and SDRs and IDP-EDL can achieve stable performance on datasets with different ratios of LDRs and SDRs. More importantly, IDP-EDL can reach or even surpass state-of-the-art performance than other existing predictors on independent test sets. IDP-EDL is available at https://github.com/joestarXjx/IDP-EDL.

## Introduction

Intrinsically disordered proteins (IDPs) are a new class of proteins that lack a stable tertiary structure under physiological conditions while possessing essential biological functions [1]. They are widely involved in important physiological processes [1–4] such as transcription and translation regulation, cellular signal transduction, and protein modification. Some diseases are also related to IDPs, such as cancer [2] and Alzheimer’s disease [5]. Therefore, accurate identification of IDPs is an essential task for understanding the structures and functions of disordered proteins. Experimental techniques for identifying IDPs include X-ray crystallography, nuclear magnetic resonance, circular dichroism [6, 7], etc. However, the above methods are not only costly and time-consuming but also encounter some unsolvable technical difficulties. Different computational methods have been established to address these challenges.

Several databases of experimentally confirmed IDPs have been established in recent years, including MobiDB [8] and Disprot [9], offering an opportunity to train reliable computational models. Effective protein embedding information and powerful algorithms are indispensable for accurately predicting intrinsically disordered regions (IDRs) within proteins. Protein sequence embedding should be extracted and encoded as numerical vectors for use in machine learning and deep learning predictors. During the past decade, the *de facto* standard in disordered protein prediction has been the use of predefined rules about properties that encapsulate evolutionary relationships between proteins or the physicochemical properties of amino acids [10]. For example, the Position-Specific Scoring Matrix [11], an evolutionary information from multiple sequence alignments (MSAs), serves as input for machine learning algorithms.

IDRs can be classified into long disordered regions (LDRs) and short disordered regions (SDRs) based on their different lengths. LDRs are typically defined as disordered regions exceeding 30 residues in length, whereas SDRs are those within 30 residues [12]. Previous studies have shown that LDRs and SDRs have different characteristics [12–15]. For example, K, E, and P are enriched in LDRs, whereas G and N are underrepresented. In contrast, G and D are preferred in SDRs, but I is not preferred [12]. To address the differences between LDRs and SDRs, length-dependent predictors were proposed. Like general predictors for predicting IDRs, MSA-based embedding information serves as input for length-dependent predictors. The difference is that length-dependent predictors specialized for the prediction of LDRs or SDRs are trained and optimized by the LDRs or SDRs data with the same algorithm, such as POODLE-L [16], POODLE-S [17], SPINE-D [12], IDP-FSP [18], IDP-Seq2Seq [19], etc. Although these length-dependent predictors have noticed differences between LDRs and SDRs, it is difficult to extract their different patterns using the same features and algorithms [20].

In recent years, inspired by the success of large language models in natural language processing [21, 22], several protein language models (pLMs) have been developed, such as ESM [23], TAPE-Transformer [24], ProtTrans [25], ProteinBERT [26], etc. pLMs learn the implicit biochemical properties, secondary and tertiary structures, and inherent functional rules in protein sequences from large data sets without any experimental annotation [27]. The information extracted by the pLMs can be easily transferred to any protein-related prediction task [28]. Protein methods that input embeddings from pLM have reached or even surpassed state-of-the-art performance in IDPs prediction tasks [29]. For example, LMDisorder [30] extracted static protein representations from the last hidden layer of pLM and was shown to be as good as other state-of-the-art methods.

Here, we propose IDP-EDL based on ensemble deep learning. It consists of three component task-specific predictors addressing the length-dependent problem in disordered regions in a three-level architecture: at the first level, a pLM encoder is employed to produce the sequence embedding information; at the second level, embeddings are inputted into different deep learning algorithms to extract different features of LDRs and SDRs; at the third level, a meta predictor fuses the three task-specific predictors’ outputs. Typically, pLM-based prediction methods utilize static, pretrained embeddings extracted from the pLM’s last hidden layer without changing its weights [30, 31]. Here, we employed Low Rank Adaptation (LoRA) [32], a particular version of a more general method known as Parameter Efficient Fine-Tuning. Injecting trainable low-rank matrices into each layer of the encoder, each component predictor was applied task-specific supervised fine-tuning. To improve the performance, stability, and generalization capabilities of the model, IDP-EDL utilized the meta-learning [33] method to combine the strengths of three task-specific models. The experimental results indicated that IDP-EDL reached or even surpassed state-of-the-art performance on different datasets and achieved stable performance on datasets with different ratios of LDRs and SDRs.

## Materials and methods

### Datasets

The dataset $S$, as shown in Table 1, used to train our model in this study is obtained from previous studies [12, 19, 34], and contain 4229 protein sequences, where the sequence similarity between any two proteins is ¡25$\%$. $S$ can be represented as


(1) \begin{align*}& S = S_{1}\cup S_{2},\end{align*}

**Table 1:** Statistical information of training dataset

	Residue level		Protein level	
Dataset	Disordered residue (percent)	Ordered residue (percent)	LD(percent)^a^	SD(percent)^b^
$S$	103 252 (10.0$\%$)	933 382 (90.0$\%$)	486 (11.5$\%$)	3743 (88.5$\%$)
$S_{1}$	74 170 (10.1$\%$)	656 634 (89.9$\%$)	342 (11.4$\%$)	2658 (88.6$\%$)
$S_{2}$	29 082 (9.5$\%$)	276 748 (90.5$\%$)	144 (11.7$\%$)	1085 (88.3$\%$)
$S^{LD}_{1}$	35 469 (30.0$\%$)	82 779 (70.0$\%$)	342 (100.0$\%$)	0 (0.0$\%$)
$S^{SD}_{1}$	38 701 (6.3$\%$)	573 855 (93.7$\%$)	0 (0.0$\%$)	2658 (100.0$\%$)

^a^ The protein with at least one LDR. ^b^ The protein with at least one SDR without LDR.

where $S_{1}$ containing 3000 proteins is selected for training the task-specific predictors and cross validation, while $S_{2}$ is employed for training the meta predictor and cross validation. The task-specific predictors are applied task-specific supervised fine-tuning and optimized by the LDRs or SDRs data. Thus, we further divide the dataset $S_{1}$ into $S^{LDR}_{1}$ and $S^{SDR}_{1}$, the first containing proteins with at least one LDR, while the second containing proteins with at least one SDR but without LDRs. $S_{1}$ can be formatted as


(2) \begin{align*}& S_{1} = S^{LD}_{1}\cup S^{SD}_{1}\end{align*}

We evaluate the model performance with other methods on three widely used independent test datasets: MXD494 [35], SL329 [36], and DISORDER723 [37]. To test the stability of IDP-EDL on datasets with different ratios of LDRs and SDRs, we construct the MSD dataset combining these three datasets and two new datasets, Disprot504 [20] and CASP [38]. In the MSD, we removed the protein sequences with a residue count greater than 2000. Table 2 lists the statistical information for all independent test datasets.

**Table 2:** Statistical information of independent test datasets

	Residue level		Protein level	
Dataset	Disordered residue (percent)	Ordered residue (percent)	LD(percent)^a^	SD(percent)^b^
MXD494	44 087 (22.4$\%$)	152 414 (77.6$\%$)	243 (49.2$\%$)	251 (50.8$\%$)
SL329	39 544 (42.4$\%$)	51 292 (57.6$\%$)	231 (70.2$\%$)	98 (29.8$\%$)
DISORDER723	13 526 (6.3$\%$)	201 703 (93.7$\%$)	56 (7.7$\%$)	667 (92.3$\%$)
MSD	166 001 (18.6$\%$)	728 639 (81.4$\%$)	1022(45.6$\%$)	1221 (54.4$\%$)

^a^ The protein with at least one LDR. ^b^ The protein with at least one SDR without LDR.

### Protein sequence embeddings

The extraction of numerical features from protein sequences is necessary before they can be utilized by machine learning or deep learning algorithms. pLMs trained on massive amounts of sequence data can encode protein sequences into dense, continuous vector representations, capturing complex structural and functional information.

In our work, we leveraged the pretrained pLM ProtT5-XL-UniRef50 [25], which we refer to as ProtT5, to extract the sequence embedding. This pretrained model undergoes self-supervised learning on 45 million sequences within the UniRef50 [39] dataset in a T5 architecture [40], employing enhanced masked language modeling. The ProtT5 encoder utilizes a 24-layer transformer encoder architecture with a parameter size of 1200M. The embedding information, more precisely the value describing the last hidden layers, is $n \times 1024$ matrix, where $n$ is the sequence length. The embedding serves as input for the subsequent layers.

### The architecture of IDP-EDL

**Figure 1:**
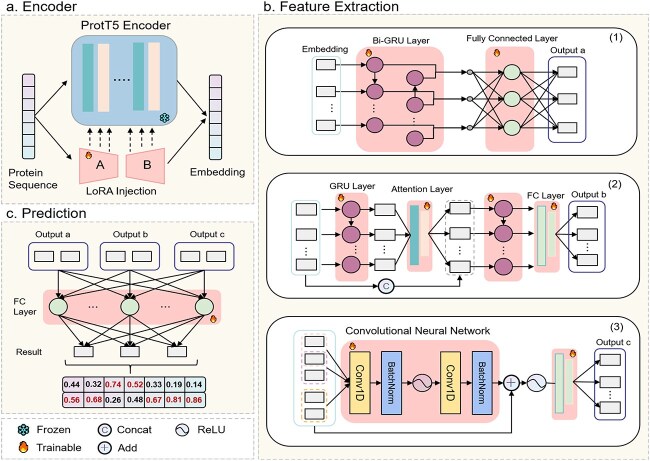
The three-layer architecture of IDP-EDL, (a) ProtT5 encoder with LoRA is used to generate the sequence embedding; (b) different deep learning algorithms are employed to capture different features between different disordered regions; (c) a meta predictor calculate final prediction results.

Figure 1 shows IDP-EDL in a three-level architecture. At the first level, the protein embeddings are extracted from the last hidden layer of the ProtT5 encoder. At the second level, embeddings are entered into the different feature extraction modules using different deep learning algorithms to capture the specific protein patterns with LDR or SDR. At the third level, a meta predictor integrates the outputs of three component predictors to produce the final prediction results.

#### PLM encoder

Formally, a protein sequence can be represented as $S =a_{1},a_{2},...,a_{n}$, where $a_{i}$ is the residue at position $i$ and $n$ is the length of the sequence. Using a pretrained embedding layer, the discrete residue-level vector $S$ is transformed into a continuous matrix. The sequence encoder takes the input $x$ and then obtains the embedding information of the protein $X \in \mathbb{R}^{n \times d} =x_{1},x_{2},...,x_{n}$, where $n$ is the sequence length and $d$ is the embedding size.

As shown in Fig. 1, LoRA [32] is implemented by injected low-rank training matrices into each layer of the transformer architecture. The encoder layer $l$ takes the residue-level embeddings $X_{l}$ from previous layer and outputs the residue-level $X_{l+1}$ embeddings in the next layer:


(3) \begin{align*}& X_{l+1} = (W_{0}+\Delta W)X_{l},\end{align*}

where $W_{0} \in \mathbb{R}^{n \times d}$ represents the pretrained weight matrix for layer $l$ and $\Delta W \in \mathbb{R}^{n \times d}$ is the trainable matrix. Let two trainable low-rank $A \in \mathbb{R}^{n \times r}$ and $B \in \mathbb{R}^{r \times d}$ ($r \ll \min (n, d)$) replace the matrix $\Delta W \in \mathbb{R}^{n \times d}$; the embeddings can be represented as


(4) \begin{align*}& X_{l+1} = (W_{0}+BA)X_{l}\end{align*}

#### Feature extraction

To capture the hidden feature associated with the length dependence between residues, we constructed three task-specific predictors, IDP-EDL-S, IDP-EDL-L, and IDP-EDL-G, using different deep learning algorithms.

In IDP-EDL-S, the embeddings extracted from the ProtT5 encoder are inputted into 1D convolutional neural networks [41, 42] to capture the local dependencies between residues in SDRs. In IDP-EDL-L, a bidirectional Gate Recurrent Unit (Bi-GRU) [43] layer is used to extract long-range dependencies among residues in LDRs. The sequence embedding, $X = {x_{1},x_{2},x_{3},...,x_{n}}$, is fed into the Bi-GRU layer, which returns the hidden state vectors of the sequence, $H = {h_{1},h_{2},h_{3},...,h_{n}}$, which can be calculated as


(5) \begin{align*}& \begin{aligned} h_{i} &= \text{Bi-GRU}(x_{i}) \\ &= \overrightarrow{\text{GRU}}(x_{i}) \oplus \overleftarrow{\text{GRU}}(x_{i}) \\ &= (\overrightarrow{\mathbf{h}}_{i} \oplus \overleftarrow{\mathbf{h}}_{i}), \end{aligned}\end{align*}

where $i$ is the time step of predicting the $i$th residue.

In IDP-EDL-G, the embeddings are inputted into the first GRU [43] layer to capture the long-term dependency information. Then, we utilized the attention mechanism [44] to dynamically focus on different regions of the input sequence. The compatibility score $e_{j}$ is represented as


(6) \begin{align*}& e_{j} = V \tanh(W_{k} h_{j} + W_{q} X),\end{align*}

where $W_{k}$ and $W_{q}$ are trainable weight matrices, and $V$ is a trainable weight vector. $h_{j}$ represents the hidden state vector at time step $j$ output by the first GRU layer, and $X$ denotes the sequence embeddings. The attention weight $alpha_{j}$ is calculated using the softmax function. The context vector $attn_{j}$ can be calculated as the weighted sum of the attention weights and the input sequence:


(7) \begin{align*} & \alpha_{j} = \frac{\exp(e_{j})}{\sum_{j=1} \exp(e_{j})}; \end{align*}

(8) \begin{align*} & attn_{j} = \sum_{i=1} \alpha_{j} x_{i}, \end{align*}

where $x_{i}$ is the embedding information at residue $i$. The context vector $attn_{j}$ and $x_{i}$ is concatenated and then fed into the second GRU layer.

#### Prediction layer

The outputs of three task-specific predictors, denoted as matrices $a$, $b$, and $c$, are $n \times 2$ matrices. The meta predictor is composed of a fully connected layer. We concatenate $a$, $b$, and $c$ into an $n \times 6$ matrix and feed it into the meta predictor to generate the final prediction of residues [45]. The result is an $n \times 2$ matrix, where each row of the output matrix represents the prediction results for ordered or disordered, and each column corresponds to the predicted score. Within a column, the higher predicted score is the prediction result for the target residue.

### Implementation details

Our training process is divided into two stages. First, task-specific predictors were constructed and task-specific supervised fine-tuning was applied for short, long, and generic disordered regions. We download the Huggingface model checkpoint of ProtT5 and use it to initialize the weighs of the encoder of the task-specific predictors. We utilized LoRA, freezing the pretrained weights in the ProtT5 encoder and injecting trainable low-rank matrices into each layer of the transformer architecture, to train up to 40 epochs. Here, we set the rank to 4 according to Equation 4, and thus the number of trainable parameters of the model is reduced from 1200M to 2500K. During the training of task-specific predictors, we employed five-fold cross-validation to ensure robust performance evaluation. Each fold was used to validate the model while the remaining folds were used for training, and the final performance metrics were averaged across all folds.

Secondly, a meta predictor was trained up to 40 epochs to integrate three task-specific predictors into the final predictor model. Similarly, five-fold cross-validation was applied during the training of the meta predictor to evaluate its generalization ability and stability. This approach allowed us to assess the meta predictor’s performance on different subsets of the data. Throughout all epochs, to prevent overfitting, we applied early stopping and added Dropout layers to our deep learning network. The dropout rate was set to 0.2. We used the Adam optimizer [46] with a learning rate of $3 \times 10^{-4}$. The binary cross-entropy loss function was utilized to optimize the model’s parameters, leading to better performance. We implemented the proposed model with the Trainer class in the Huggingface transformers library [47].

### Evaluation metrics

Five performance measures were used to access the performance of different predictors, including Sensitivity (Sn), Specificity (Sp), Matthew’s correlation coefficient (MCC), Balanced Accuracy (BACC), and Area under the ROC curve (AUC) [12, 38, 48]. These metrics are calculated as


(9) \begin{align*}& \begin{cases} S_{n} = \frac{TP}{TP + FN}\\[3pt] S_{P} = \frac{TN}{TN + FP}\\[4pt] BACC = \frac{1}{2}(Sn + Sp) \\[4pt] MCC = \frac{TP \times TN - FP \times FN}{\sqrt{(TP + FP)(TP + FN)(TN + FP)(TN + FN)}}, \end{cases}\end{align*}

where TP, TN, FP, and FN are the respective numbers of true positives, true negatives, false positives, and false negatives, respectively.

## Results and discussion

###  

#### LoRA boosted disorder prediction

Research indicates that fine-tuning the parameters of large language models in natural language processing is beneficial for downstream predictive performance [29]. Here, we evaluated the impact of supervised fine-tuning on disordered protein prediction tasks by adding a simple fully connected layer as the prediction head on top of the ProtT5 encoder. We compared fine-tuning methods including full fine-tuning, shallow fine-tuning [49], deep fine-tuning [49], and LoRA with the method without fine-tuning. The method without fine-tuning only trained the prediction head with freezing the parameters of the ProtT5 encoder, whereas the fine-tuning methods applied supervised training to both the encoder and prediction head. In ablation experiments, shallow fine-tuning only updated the first two layers of the pretrained network, as well as deep fine-tuning, where the last two layers were also subject to parameter updates.

**Figure 2:**
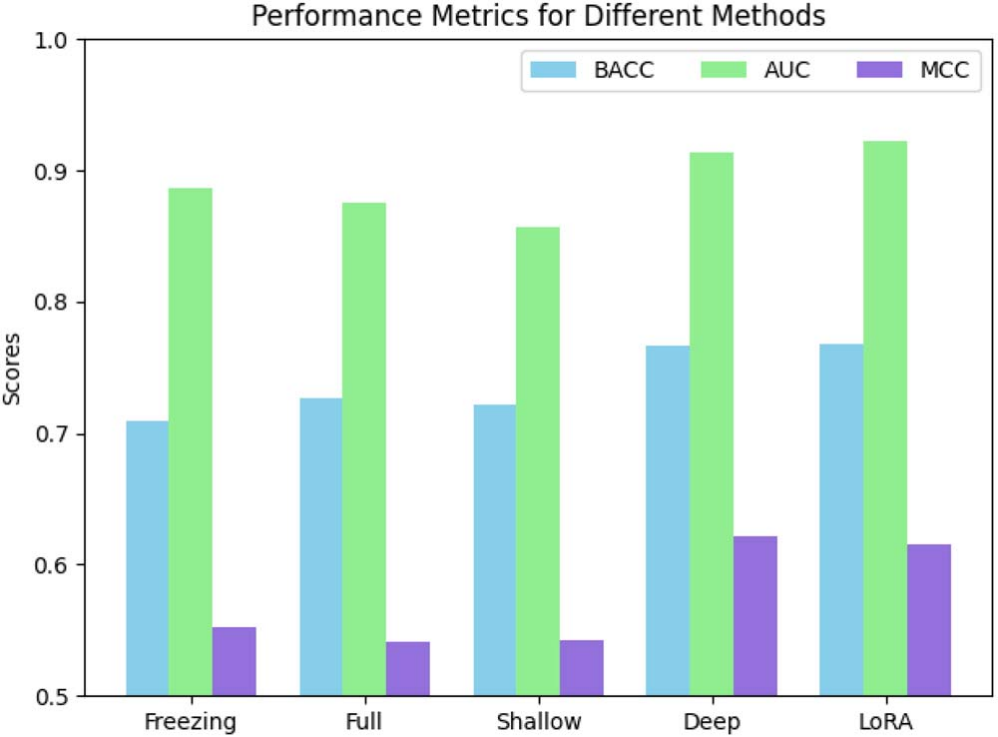
Performance metrics for different methods on dataset $S_{2}$.

Far a fair test, all these methods were trained on dataset $S_{1}$ and tested on dataset $S_{2}$. Figure 2 illustrates the performance metrics for the aforementioned methods on dataset $S_{2}$. We note that full and shallow fine-tuning do not improve the model, while deep fine-tuning and LoRA can boost disordered protein prediction. These indicate that full fine-tuning and shallow fine-tuning may cause the model to forget previously learned protein knowledge, while deep fine-tuning and LoRA can adapt to the specific knowledge of disordered proteins, better capturing embedding information for disordered proteins. Notably, compared with the other three fine-tuning methods full fine-tuning, shallow fine-tuning, and deep fine-tuning with trainable parameters of 1200M, 100M, and 100M, respectively, LoRA has 2500K trainable parameters and is the best-performing method. Thus, LoRA is the most beneficial among these four fine-tuning methods for disordered protein prediction tasks.

###  

#### Ensemble of task-specific predictors can achieve stable predictive performance

An IDP may contain both LDRs and SDRs. Among these, LDRs are relatively easier to identify. SDRs are short and important regions that are discretely distributed, and computational methods may easily filter them out. To capture the different patterns of LDRs and SDRs, the component predictors were built based on different deep learning algorithms and applied task-specific supervised fine-tuning.

**Figure 3:**
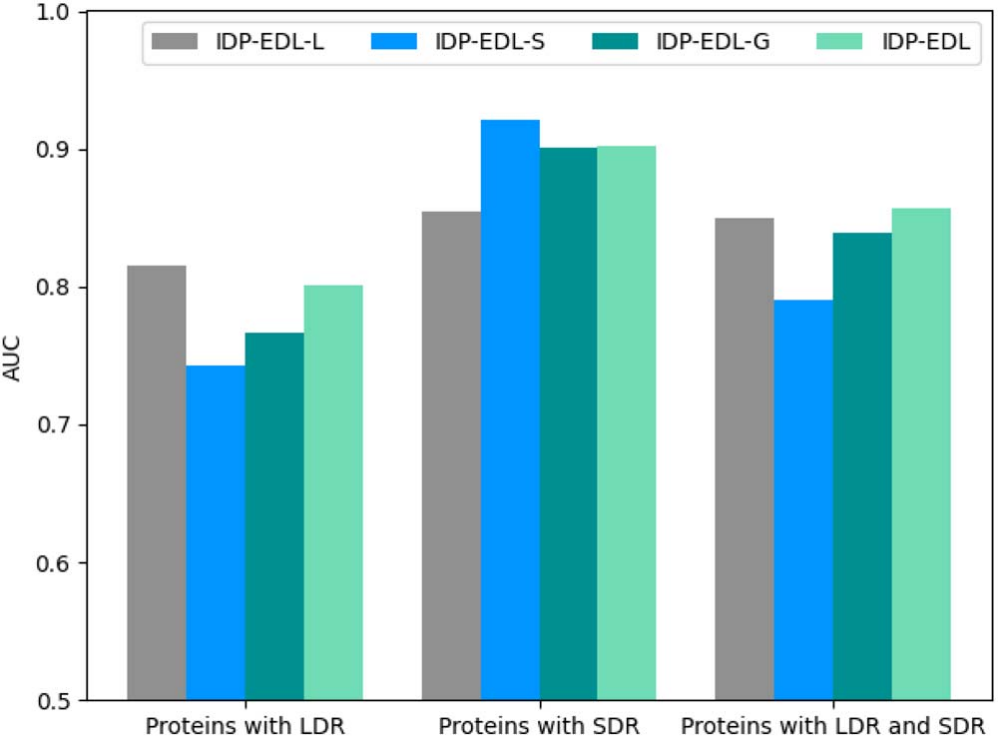
The AUC values of IDP-EDL-L, IDP-EDL-S, IDP-EDL-G, and IDP-EDL on datasets $MSD_{LD}$, $MSD_{SD}$, and $MSD$.

We conducted three sets of experiments, with three task-specific predictors tested on $S_{2}^{LD}, S_{2}^{SD}, and S_{2}$. $S_{2}^{LD}$ consists of proteins that include at least one LDR; $S_{2}^{SD}$ comprises proteins with one SDR and without LDR. $S_{2}$ is formed by combining $S_{2}^{LD}$ and $S_{2}^{SD}$. As shown in Table 3, we can see the following: the three task-specific predictors achieve the best performance on the test datasets with the same disordered type as their training sets, indicating that task-specific supervised fine-tuning can improve predictive performance for disordered regions with specific length. Furthermore, we divided $MSD$ into $MSD_{LD}$ and $MSD_{SD}$ as the same as the aforementioned method. The AUC values of three task-specific predictors and IDP-EDL on the datasets $MSD_{LD}$, $MSD_{SD}$ and $MSD$ are shown in Fig. 3. We can see that IDP-EDL achieves stable performance on all three datasets, indicating that IDP-EDL integrates the three task-specific predictors in a complementary manner.

**Table 3:** Performance of IDP-EDL-G, IDP-EDL-L, and IDP-EDL-S on test datasets

Test set	Predictor	Sn	Sp	BACC	MCC	AUC
$S_{2}^{LD}$	IDP-EDL-G	0.636	0.939	0.787	0.620	0.886
	IDP-EDL-L	**0.718**	0.940	**0.829**	**0.685**	**0.921**
	IDP-EDL-S	0.427	**0.979**	0.703	0.536	0.824
$S_{2}^{SD}$	IDP-EDL-G	0.560	**0.980**	0.770	0.593	0.934
	IDP-EDL-L	0.580	0.913	0.747	0.373	0.859
	IDP-EDL-S	**0.604**	0.979	**0.792**	**0.608**	**0.937**
$S_{2}$	IDP-EDL-G	0.593	0.975	**0.784**	**0.618**	**0.923**
	IDP-EDL-L	**0.643**	0.916	0.779	0.476	0.882
	IDP-EDL-S	0.528	**0.979**	0.754	0.586	0.885

###  

#### Comparison with other methods on independent test sets

To comprehensively evaluate the performance of IDP-EDL, we compared it with other existing methods on three commonly used independent test sets, including MXD494 [35], SL329 [36], and DISORDER723 [37], each containing obviously ratios of LDRs and SDRs, as shown in Table 2. To fairly evaluate the performance of different methods and avoid overestimating the performance of IDP-EDL, we removed sequences with similarity exceeding 25$\%$ between between the independent datasets and the training dataset. IDP-EDL was then retrained using the nonredundant training dataset and subsequently applied to predict the independent datasets.

The prediction results are listed in Tables 4, 5, and 6, from which we can see the following: (i) some predictors show significant performance variation between different test sets. For example, the AUC values of MFDp and MD rank fourth on the MXD494 datasets, and ninth and 10th on the SL329 datasets, respectively. This indicates that these predictors do not achieve stable performance on datasets with different ratios of LDRs and SDRs; (ii) some predictors improve the Sn value by a decrease in Sp value, but the AUC value does not increase significantly, indicating that these predictors ignore the difference between LDRs and SDRs; (iii) IDP-EDL achieved or even exceeded state-of-the-art performance with the highest AUC and MCC values on each independent test set, indicating that IDP-EDL can achieve more stable performance than other existing predictors on independent test sets, with a significant improvement in the BACC, MCC, and AUC values without a decrease in Sn and Sp values.

**Table 4:** Performance of various methods on MXD494

Predictor	Sn	Sp	BACC	MCC	AUC	Rank		
						AUC	BACC	MCC
IDP-EDL	0.679	0.843	**0.761**	**0.488**	**0.837**	1	2	1
DeepIDP-2L [20]	0.737	0.776	**0.757**	0.452	**0.825**	2	3	5
IDP-Seq2Seq [19]	0.743	0.791	**0.767**	**0.475**	**0.825**	2	1	2
MFDp [50]	0.746	0.768	**0.757**	0.451	0.821	4	3	6
MD [51]	0.673	0.813	0.743	0.444	0.821	4	7	7
RFPR-IDP [52]	0.749	0.758	0.754	0.442	0.821	4	5	8
SPOT-Disorder [53]	0.626	0.851	0.739	0.457	0.813	7	9	4
SPINE-D [12]	0.787	0.698	0.742	0.411	0.803	8	8	10
AUCpreD [38]	0.521	0.881	0.701	0.411	0.800	9	15	10
DISOPRED3 [54]	0.622	0.820	0.721	0.410	0.800	9	12	12
IDP-FSP [18]	0.670	0.831	0.751	**0.465**	0.794	11	6	3
PONDER-FIT [55]	0.631	0.821	0.726	0.419	0.790	12	10	9
IUPred-long [56]	0.581	0.841	0.711	0.405	0.784	13	13	14
DISOPRED2 [57]	0.647	0.800	0.724	0.406	0.781	14	11	13
IUPred-short [56]	0.522	0.866	0.694	0.389	0.781	14	16	15
DISpro [37]	0.303	0.940	0.622	0.318	0.775	16	19	18
RONN [58]	0.664	0.754	0.709	0.368	0.764	17	14	16
Ucon [59, 60]	0.554	0.787	0.671	0.313	0.741	18	18	19
NORSnet [59, 60]	0.532	0.829	0.681	0.347	0.738	19	17	17
PROFbval [61]	0.835	0.387	0.611	0.196	0.697	20	20	20

**Table 5:** Performance of various methods on SL329

Predictor	Sn	Sp	BACC	MCC	AUC	Rank		
						AUC	BACC	MCC
IDP-EDL	0.69	0.97	**0.828**	**0.70**	**0.915**	1	2	1
DeepIDP-2L [20]	0.74	0.92	**0.830**	**0.68**	**0.904**	2	1	2
SPOT-Disorder [53]	0.65	0.96	0.805	0.65	**0.901**	3	7	4
IDP-Seq2Seq [19]	0.71	0.92	**0.822**	**0.67**	0.899	4	3	3
AUCpreD [38]	0.63	0.96	0.795	0.64	0.887	5	9	5
SPINE-D [12]	0.82	0.80	0.815	0.61	0.886	6	5	9
DISOPRED3 [54]	0.67	0.92	0.796	0.62	0.880	7	8	7
RFPR-IDP [52]	0.78	0.84	0.809	0.62	0.879	8	6	7
MFDp [50]	0.88	0.62	0.750	0.51	0.873	9	15	14
MD [51]	0.66	0.89	0.775	0.58	0.864	10	11	11
IDP-FSP [18]	0.75	0.89	0.821	0.65	0.864	10	4	6
DISOPRED2 [57]	0.69	0.90	0.795	0.59	0.858	12	9	10
DISOClust [62]	0.81	0.70	0.755	0.51	0.846	13	14	14
PONDR-FIT [55]	0.61	0.91	0.760	0.55	0.843	14	12	12
IUpred-long [56]	0.60	0.92	0.760	0.55	0.839	15	12	12
IUpred-short [56]	0.50	0.94	0.720	0.50	0.829	16	17	17
NORSnet [59, 60]	0.54	0.92	0.730	0.51	0.815	17	16	14
Ucon [59, 60]	0.59	0.81	0.700	0.42	0.779	18	18	18
PONDERVL-XT [18]	0.59	0.78	0.685	0.38	0.755	19	19	19

**Table 6:** Performance of various methods on DISORDER723

Predictor	Sn	Sp	BACC	MCC	AUC	Rank		
						AUC	BACC	MCC
IDP-EDL	0.603	0.984	**0.793**	**0.636**	**0.943**	1	1	1
DeepIDP-2L [20]	0.615	0.962	0.789	0.529	0.914	2	2	5
IDP-Seq2Seq [19]	0.618	0.955	0.787	0.511	0.906	3	3	7
AUCpreD [38]	0.580	0.974	0.777	0.564	0.914	4	4	2
DISOPRED3 [54]	0.452	0.986	0.719	0.536	0.899	5	7	3
SPOT-Disorder [34]	0.470	0.983	0.726	0.531	0.898	6	6	4
RFPR-IDP [52]	0.522	0.974	0.748	0.517	0.898	7	5	6

###  

#### IDP-EDL is insensitive with the different ratios of LDRs and SDRs

The prediction dataset always includes both LDR proteins and SDR proteins. For such a situation, we constructed the task-specific predictor IDP-EDL-G. However, the ratio between LDR proteins and SDR proteins is unknown in real-world applications. Thus, a stable performance predictor is preferred for predicting both LDR proteins and SDR proteins.

**Figure 4:**
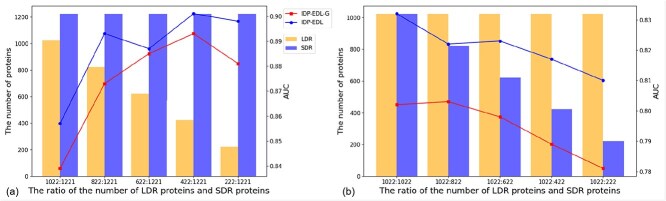
The AUC values of IDP-EDL-G and IDP-EDL evaluated on the datasets with different ratios of LDRs and SDRs.

For further evaluating the performance of IDP-EDL and IDP-EDL-G, we constructed 10 datasets with different ratios between LDR proteins and SDR proteins from the MSD dataset. The AUC values predicted by these two predictors are shown in Fig. 4, from which we can see the following:

- The AUC values predicted by IDP-EDL are higher than those predicted by IDP-EDL-G on each dataset, indicating that IDP-EDL achieves more stable performance for predicting proteins with different ratios between LDRs and SDRs.
- These two predictors tend to perform better with the increase of proteins with SDR and the decrease of proteins with LDR, which indicates that proteins with SDR can be predicted more accurately than proteins with LDR.
- IDP-EDL achieves AUC values exceeding 0.80 on all 10 datasets, indicating that the ratios between LDRs and SDRs have limited impact on IDP-EDL performance.

Different types of disordered regions have different characteristics. The task-specific predictors were constructed to capture these differences between LDR and SDR. IDP-EDL integrates these task-specific predictors in a complementary manner. Thus, IDP-EDL can achieve stable performance in predicting different ratios of LDR proteins and SDR proteins. The stable performance of IDP-EDL in predicting disordered proteins is especially useful for practical applications.

## Conclusion

The LDRs and SDRs have different characteristics. The existing predictors can be divided into two categories: general predictors and length-dependent predictors. The general predictors ignore the differences between LDRs and SDRs [34, 38, 63]. Although the length-dependent notice the differences between LDRs and SDRS, it is difficult to extract their different features by using the same features and algorithms. In this study, we proposed a predictor named IDP-EDL. It consists of three task-specific predictors in a three-layer architecture. Compared with other computational methods, it has the following advantages: (i) the predictor utilizes the pretrained model to produce sequence embeddings and does not require database searching like MSA-based predictors [19, 20]; thus, IDP-EDL is a time-saving method; (ii) other pLM-based predictors extract static, pretrained embeddings from pLM’s last hidden layer without changing its weights [30, 31]. Although they take into account the general features of proteins, they do not consider the specific features of IDPs. Thus, we employed task-specific fine-tuning based on LoRA for IDP prediction; (iii) we can use task-specific predictors IDP-EDL-L, IDP-EDL-S, and IDP-EDL-G to address the corresponding prediction tasks and use IDP-EDL to solve the unknown prediction problems, because IDP-EDL achieves stable performance with different ratios of LDRs and SDRs; (iv) IDP-EDL is available at https://github.com/joestarXjx/IDP-EDL. It can be easily deployed locally and used for IDP predictions according to the README file.

IDP-EDL is a general predictor that would be applied to solve other prediction tasks related to disordered regions. For example, the prediction of disordered lipid-binding residues in protein sequences is important to study the biological functions of IDRs [64]. Disordered lipid-binding region (DLBR) is intrinsically disordered and interact with lipids. Thus, DLBR prediction has benefited from the development of IDP predictions based on the similarities between DLBR prediction and IDP prediction. We believe that the predictors proposed in this study can contribute to the prediction tasks related to IDP.

**Key Points**

- pLM-based embeddings are utilized in this study. Compared with MSA-based embeddings, this method does not require database searching.
- IDP-EDL is an ensemble of three predictors. It can achieve stable performance with different ratios of LDRs and SDRs.
- The component predictors of IDP-EDL were applied to LoRA-based task-specific supervised fine-tuning for long, short, and generic disordered regions.
- The experimental results demonstrate that IDP-EDL can reach or even surpass state-of-the-art performance compared with other existing predictors on independent test sets.

## References

[1] Jane Dyson H, Wright PE. Intrinsically unstructured proteins and their functions. *Nat Rev Mol Cell Biol* 2005;6:197–208. 10.1038/nrm1589 15738986

[2] Iakoucheva LM, Brown CJ, David Lawson J. et al. Intrinsic disorder in cell-signaling and cancer-associated proteins. *J Mol Biol* 2002;323:573–84. 10.1016/S0022-2836(02)00969-5 12381310

[3] Piovesan D, Tabaro F, Mičetić I. et al. Disprot 7.0: a major update of the database of disordered proteins. *Nucleic Acids Res* 2017;45:D219–27. 27899601 10.1093/nar/gkw1056 PMC5210544

[4] Pang Y, Liu B. Disoflag: accurate prediction of protein intrinsic disorder and its functions using graph-based interaction protein language model. *BMC Biol* 2024;22:3. 38166858 10.1186/s12915-023-01803-y PMC10762911

[5] Uversky VN, Oldfield CJ, Keith A. et al. Intrinsically disordered proteins in human diseases: introducing the d2 concept. *Annu Rev Biophys* 2008;37:215–46. 10.1146/annurev.biophys.37.032807.125924 18573080

[6] Receveur-Bréchot V, Bourhis J-M, Uversky VN. et al. Assessing protein disorder and induced folding. *Proteins* 2006;62:24–45. 10.1002/prot.20750 16287116

[7] Konrat R . Nmr contributions to structural dynamics studies of intrinsically disordered proteins. *J Magn Reson* 2014;241:74–85. 10.1016/j.jmr.2013.11.011 24656082 PMC3985426

[8] Potenza E, Di Domenico, Walsh I. et al. Mobidb 2.0: an improved database of intrinsically disordered and mobile proteins. *Nucleic Acids Res* 2015;43:D315–20. 10.1093/nar/gku982 25361972 PMC4384034

[9] Hatos A, Hajdu-Soltész B, Monzon AM. et al. Disprot: intrinsic protein disorder annotation in 2020. *Nucleic Acids Res* 2020;48:D269–76. 10.1093/nar/gkz975 31713636 PMC7145575

[10] Unsal S, Atas H, Albayrak M. et al. Learning functional properties of proteins with language models. *Nature*. *Mach Intell* 2022;4:227–45.

[11] Wang J, Yang B, Revote J. et al. Possum: a bioinformatics toolkit for generating numerical sequence feature descriptors based on pssm profiles. *Bioinformatics* 2017;33:2756–8. 10.1093/bioinformatics/btx302 28903538

[12] Zhang T, Faraggi E, Bin Xue A. et al. Spine-d: accurate prediction of short and long disordered regions by a single neural-network based method. *J Biomol Struct Dyn* 2012;29:799–813. 10.1080/073911012010525022 22208280 PMC3297974

[13] Peng K, Radivojac P, Slobodan Vucetic A. et al. Length-dependent prediction of protein intrinsic disorder. *BMC Bioinformatics* 2006;7:1–17. 16618368 10.1186/1471-2105-7-208 PMC1479845

[14] Radivojac P, Obradovic Z, Smith DK. et al. Protein flexibility and intrinsic disorder. *Protein Sci* 2004;13:71–80. 10.1110/ps.03128904 14691223 PMC2286519

[15] Pang Y, Liu B. Idp-lm: prediction of protein intrinsic disorder and disorder functions based on language models. *PLoS Comput Biol* 2023;19:e1011657. 10.1371/journal.pcbi.1011657 37992088 PMC10699601

[16] Hirose S, Shimizu K, Kanai S. et al. Poodle-l: a two-level svm prediction system for reliably predicting long disordered regions. *Bioinformatics* 2007;23:2046–53. 10.1093/bioinformatics/btm302 17545177

[17] Shimizu K, Hirose S, Noguchi T. Poodle-s: web application for predicting protein disorder by using physicochemical features and reduced amino acid set of a position-specific scoring matrix. *Bioinformatics* 2007;23:2337–8. 10.1093/bioinformatics/btm330 17599940

[18] Liu Y, Chen S, Wang X. et al. Identification of intrinsically disordered proteins and regions by length-dependent predictors based on conditional random fields. *Mol Ther Nucleic Acids* 2019;17:396–404. 10.1016/j.omtn.2019.06.004 31307006 PMC6626971

[19] Tang Y-J, Pang Y-H, Liu B. Idp-seq2seq: identification of intrinsically disordered regions based on sequence to sequence learning. *Bioinformatics* 2020;36:5177–86. 10.1093/bioinformatics/btaa667 32702119

[20] Tang Y-J, Pang Y-H, Liu B. Deepidp-2l: protein intrinsically disordered region prediction by combining convolutional attention network and hierarchical attention network. *Bioinformatics* 2022;38:1252–60. 10.1093/bioinformatics/btab810 34864847

[21] Devlin J, Chang MW, Lee K. et al. BERT: pre-training of deep bidirectional transformers for language understanding. In: NAACL HLT 2019 – 2019 Conference of the North American Chapter of the Association for Computational Linguistics: Human Language Technologies - Proceedings of the Conference, Vol. 1, pp. 4171–86, Minneapolis, USA: Association for ComputationalLinguistics, 2019. 10.1136/bmjgh-2018-000912

[22] Vaswani A, Shazeer N, Parmar N. et al. Attention is all you need. *Advances in neural information processing systems* 2017;30:5998–8.

[23] Rives A, Meier J, Sercu T. et al. Biological structure and function emerge from scaling unsupervised learning to 250 million protein sequences. *Proc Natl Acad Sci* 2021;118:e2016239118. 33876751 10.1073/pnas.2016239118 PMC8053943

[24] Rao R, Bhattacharya N, Thomas N. et al. Evaluating protein transfer learning with tape. *Advances in neural information processing systems* 2019;32:9686–98. PMC7774645 33390682

[25] Elnaggar A, Heinzinger M, Dallago C. et al. Prottrans: toward understanding the language of life through self-supervised learning. *IEEE Trans Pattern Anal Mach Intell* 2021;44:7112–27. 10.1109/TPAMI.2021.3095381 34232869

[26] Brandes N, Ofer D, Peleg Y. et al. Proteinbert: a universal deep-learning model of protein sequence and function. *Bioinformatics* 2022;38:2102–10. 10.1093/bioinformatics/btac020 35020807 PMC9386727

[27] Ofer D, Brandes N, Linial M. The language of proteins: Nlp, machine learning & protein sequences. *Comput Struct Biotechnol J* 2021;19:1750–8. 10.1016/j.csbj.2021.03.022 33897979 PMC8050421

[28] Wu L, Huang Y, Lin H. et al. A survey on protein representation learning: retrospect and prospect arXiv preprint arXiv:2301.00813. 2022.

[29] Schmirler R, Heinzinger M, Rost B. Fine-tuning protein language models boosts predictions across diverse tasks. *Nat Commun* 2024;15:7407. 39198457 10.1038/s41467-024-51844-2 PMC11358375

[30] Song Y, Yuan Q, Chen S. et al. Fast and accurate protein intrinsic disorder prediction by using a pretrained language model. *Brief Bioinform* 2023;24:bbad173. 37204193 10.1093/bib/bbad173

[31] Yang Z, Wang Y, Ni X. et al. Deepdrp: prediction of intrinsically disordered regions based on integrated view deep learning architecture from transformer-enhanced and protein information. *Int J Biol Macromol* 2023;253:127390. 10.1016/j.ijbiomac.2023.127390 37827403

[32] Hu EJ, Shen Y, Wallis P. et al. Lora: low-rank adaptation of large language models arXiv preprint arXiv:2106.09685. 2021.

[33] Mohammed A, Kora R. A comprehensive review on ensemble deep learning: opportunities and challenges. *J King Saud Univ Comput Inf Sci* 2023;35:757–74. 10.1016/j.jksuci.2023.01.014

[34] Hanson J, Paliwal KK, Litfin T. et al. Spot-disorder2: improved protein intrinsic disorder prediction by ensembled deep learning. *Genomics Proteomics Bioinf* 2019;17:645–56. 10.1016/j.gpb.2019.01.004 PMC7212484 32173600

[35] Peng Z-L, Kurgan L. Comprehensive comparative assessment of in-silico predictors of disordered regions. *Curr Protein Pept Sci* 2012;13:6–18. 10.2174/138920312799277938 22044149

[36] Sirota FL, Ooi H-S, Gattermayer T. et al. Parameterization of disorder predictors for large-scale applications requiring high specificity by using an extended benchmark dataset. *BMC Genomics* 2010;11:1–17. 20158872 10.1186/1471-2164-11-S1-S15 PMC2822529

[37] Cheng J, Sweredoski MJ, Baldi P. Accurate prediction of protein disordered regions by mining protein structure data. *Data Min Knowl Discovery* 2005;11:213–22. 10.1007/s10618-005-0001-y

[38] Wang S, Ma J, Jinbo X. Aucpred: proteome-level protein disorder prediction by auc-maximized deep convolutional neural fields. *Bioinformatics* 2016;32:i672–9. 10.1093/bioinformatics/btw446 27587688 PMC5013916

[39] Suzek BE, Huang H, McGarvey P. et al. Uniref: comprehensive and non-redundant uniprot reference clusters. *Bioinformatics* 2007;23:1282–8. 10.1093/bioinformatics/btm098 17379688

[40] Raffel C, Shazeer N, Roberts A. et al. Exploring the limits of transfer learning with a unified text-to-text transformer. *J Mach Learn Res* 2020;21:1–67.34305477

[41] He K, Zhang X, Ren S. et al. Deep residual learning for image recognition. In: Proceedings of the IEEE conference on computer vision and pattern recognition. 2016, p. 770–8.

[42] Guo B, Zhang C, Liu J. et al. Improving text classification with weighted word embeddings via a multi-channel textcnn model. *Neurocomputing* 2019;363:366–74. 10.1016/j.neucom.2019.07.052

[43] Chung J, Gulcehre C, Cho KH. et al. Empirical evaluation of gated recurrent neural networks on sequence modeling. Advances in neural information processing systems, MIT Press, Montreal Canada. 10.1109/TVCG.2013.272

[44] Bahdanau D, Cho K, Bengio Y. Neural machine translation by jointly learning to align and translate. In International Conference on Learning Representations, pp. 1–9. San Diego, CA, 2015.

[45] Cao Y, Geddes TA, Yang JYH. et al. Ensemble deep learning in bioinformatics. *Nat Mach Intell* 2020;2:500–8. 10.1038/s42256-020-0217-y

[46] Kinga D, Adam JB. et al. A method for stochastic optimization. In International Conference on Learning Representations, Vol. 5, p. 6. San Diego, CA, 2015.

[47] Shen Y, Kaitao Song X, Tan DL. et al. Hugginggpt: solving ai tasks with chatgpt and its friends in hugging face. *Advances in Neural Information Processing Systems* 2024;36:38154–80.

[48] Zhang W, Wei H, Zhang W. et al. Multiple types of disease-associated rnas identification for disease prognosis and therapy using heterogeneous graph learning. *Sci China Inf Sci* 2024;67:189103.

[49] Guo Y, Shi H, Kumar A. et al. Spottune: Transfer learning through adaptive fine-tuning. In: Proceedings of the IEEE/CVF conference on computer vision and pattern recognition. 2019, p. 4805–14.

[50] Mizianty MJ, Stach W, Chen K. et al. Improved sequence-based prediction of disordered regions with multilayer fusion of multiple information sources. *Bioinformatics* 2010;26:i489–96. 10.1093/bioinformatics/btq373 20823312 PMC2935446

[51] Schlessinger A, Punta M, Yachdav G. et al. Improved disorder prediction by combination of orthogonal approaches. *PloS One* 2009;4:e4433. 10.1371/journal.pone.0004433 19209228 PMC2635965

[52] Liu Y, Wang X, Liu B. Rfpr-idp: reduce the false positive rates for intrinsically disordered protein and region prediction by incorporating both fully ordered proteins and disordered proteins. *Brief Bioinform* 2021;22:2000–11. 10.1093/bib/bbaa018 32112084 PMC7986600

[53] Hanson J, Yang Y, Paliwal K. et al. Improving protein disorder prediction by deep bidirectional long short-term memory recurrent neural networks. *Bioinformatics* 2017;33:685–92. 10.1093/bioinformatics/btw678 28011771

[54] Jones DT, Cozzetto D. Disopred3: precise disordered region predictions with annotated protein-binding activity. *Bioinformatics* 2015;31:857–63. 10.1093/bioinformatics/btu744 25391399 PMC4380029

[55] Xue B, Dunbrack RL, Williams RW. et al. Pondr-fit: a meta-predictor of intrinsically disordered amino acids. *Biochim Biophys Acta Proteins Proteomics* 2010;1804:996–1010. 10.1016/j.bbapap.2010.01.011 PMC2882806 20100603

[56] Dosztányi Z, Csizmok V, Tompa P. et al. Iupred: web server for the prediction of intrinsically unstructured regions of proteins based on estimated energy content. *Bioinformatics* 2005;21:3433–4. 10.1093/bioinformatics/bti541 15955779

[57] Ward JJ, Sodhi JS, McGuffin LJ. et al. Prediction and functional analysis of native disorder in proteins from the three kingdoms of life. *J Mol Biol* 2004;337:635–45. 10.1016/j.jmb.2004.02.002 15019783

[58] Yang ZR, Thomson R, McNeil P. et al. Ronn: the bio-basis function neural network technique applied to the detection of natively disordered regions in proteins. *Bioinformatics* 2005;21:3369–76. 10.1093/bioinformatics/bti534 15947016

[59] Schlessinger A, Liu J, Rost B. Natively unstructured loops differ from other loops. *PLoS Comput Biol* 2007;3:e140. 10.1371/journal.pcbi.0030140 17658943 PMC1924875

[60] Schlessinger A, Punta M, Rost B. Natively unstructured regions in proteins identified from contact predictions. *Bioinformatics* 2007;23:2376–84. 10.1093/bioinformatics/btm349 17709338

[61] Schlessinger A, Yachdav G, Rost B. Profbval: predict flexible and rigid residues in proteins. *Bioinformatics* 2006;22:891–3. 10.1093/bioinformatics/btl032 16455751

[62] McGuffin LJ . Intrinsic disorder prediction from the analysis of multiple protein fold recognition models. *Bioinformatics* 2008;24:1798–804. 10.1093/bioinformatics/btn326 18579567

[63] Liu Y, Wang X, Liu B. Idp–crf: intrinsically disordered protein/region identification based on conditional random fields. *Int J Mol Sci* 2018;19:2483. 10.3390/ijms19092483 30135358 PMC6164615

[64] Katuwawala A, Zhao B, Kurgan L. Disolippred: accurate prediction of disordered lipid-binding residues in protein sequences with deep recurrent networks and transfer learning. *Bioinformatics* 2022;38:115–24. 10.1093/bioinformatics/btab640 34487138

